# Current evidence for avoidant restrictive food intake disorder: Implications for clinical practice and future directions

**DOI:** 10.1002/jcv2.12160

**Published:** 2023-04-03

**Authors:** Tanith Archibald, Rachel Bryant‐Waugh

**Affiliations:** ^1^ Maudsley Centre for Child and Adolescent Eating Disorders Michael Rutter Centre Maudsley Hospital London UK; ^2^ Department of Child and Adolescent Psychiatry Institute of Psychiatry, Psychology and Neuroscience Kings College London London UK

**Keywords:** ARFID, assessment, clinical practice, narrative review, prevalence, service delivery, treatment

## Abstract

**Background:**

ARFID (avoidant restrictive food intake disorder) is a relatively new diagnostic term covering a number of well‐recognised, clinically significant disturbances in eating behaviour unrelated to body weight/shape concerns. Its phenotypic heterogeneity combined with much about the condition remaining unknown, can contribute to uncertainties about best practice. While other reviews of the evidence base for ARFID exist, few specifically target health care professionals and implications for clinical practice.

**Methods:**

A narrative review was conducted to synthesise the findings of ARFID papers in scientific journals focussing on four key areas relevant to clinical practice: prevalence, assessment and characterisation of clinical presentations, treatment, and service delivery. Freely available online databases were searched for case studies and series, research reports, review articles, and meta‐analyses. Findings were reviewed and practice implications considered, resulting in proposed clinical recommendations and future research directions.

**Results:**

We discuss what is currently known about the four key areas included in this review. Based on available evidence as well as gaps identified in the literature, recommendations for clinical practice are derived and practice‐related research priorities are proposed for each of the four of the areas explored.

**Conclusion:**

Prevalence studies highlight the need for referral and care pathways to be embedded across a range of health care services. While research into ARFID is increasing, further studies across all areas of ARFID are required and there remains a pressing need for guidance on systematic assessment, evidence‐based management, and optimal service delivery models. Informed clinical practice is currently predominantly reliant on expert consensus and small‐scale studies, with ongoing routine clinical data capture, robust treatment trials and evaluation of clinical pathways all required. Despite this, a number a positive practice points emerge.

## Graphical abstract

This paper reviews of the evidence base for ARFID specifically targeting health care professionals and implications for clinical practice. A narrative review was conducted to synthesise current evidence on four key areas relevant to clinical practice: prevalence, assessment and characterisation of clinical presentations, treatment, and service delivery. Findings are reviewed and practice implications considered, resulting in proposed clinical recommendations and future research directions.

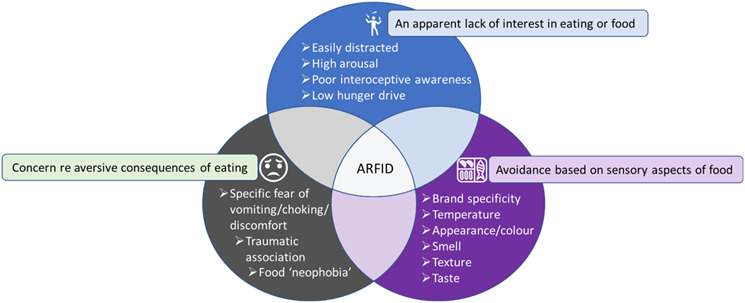

**Key points**

- ARFID is a heterogeneous diagnostic category, with multidisciplinary and multi‐modal assessment and treatment approaches recommended.
- Further work on systematic assessment and characterisation of clinical presentations will facilitate ARFID profile recognition and support development of optimal treatment planning.
- Recommended ARFID management includes targeting maintaining factors of the eating disturbance through a psychological treatment intervention alongside medical, dietetic and other indicated interventions tailored to address domains of impact and risk specific to the individual.
- Optimal service provision requires integrated referral and care pathways, which may involve networks of professionals and services, to ensure timely, appropriate care.
- Analysis of routinely captured clinical data can helpfully complement research studies to support evidence‐based clinical practice and patient‐centred care.

## INTRODUCTION

Avoidant restrictive food intake disorder (ARFID) is a diagnostic term for a disturbance in eating behaviour that results in failure to meet appropriate nutritional and/or energy needs leading to physical impairment, or results in psychosocial impairment. Many people with ARFID experience impairment across both domains. While children, young people and adults presenting to clinical settings with symptoms of ARFID is not a new phenomenon, terminology, understanding, and characterisation of the condition have advanced since its inclusion in the Diagnostic and Statistical Manual of Mental Disorders (DSM‐5; American Psychiatric Association [APA], 2013) and the World Health Organization's International Classification of Diseases (ICD‐11; World Health Organization, 2019). ARFID now sits in the same category of feeding and eating disorders as anorexia nervosa (AN) and bulimia nervosa (BN), distinguished from these eating disorders by the absence of body shape or weight concerns as a key factor maintaining the eating disturbance. Although the main distinctions between ARFID and other eating disorders are clear, its development and clinical features can vary considerably between individuals. This phenotypic heterogeneity, with diverse symptomatic profiles typically maintained by interwoven contributing factors, combined with much about the condition remaining unknown, can contribute to uncertainties related to clinical practice.

**FIGURE 1:**
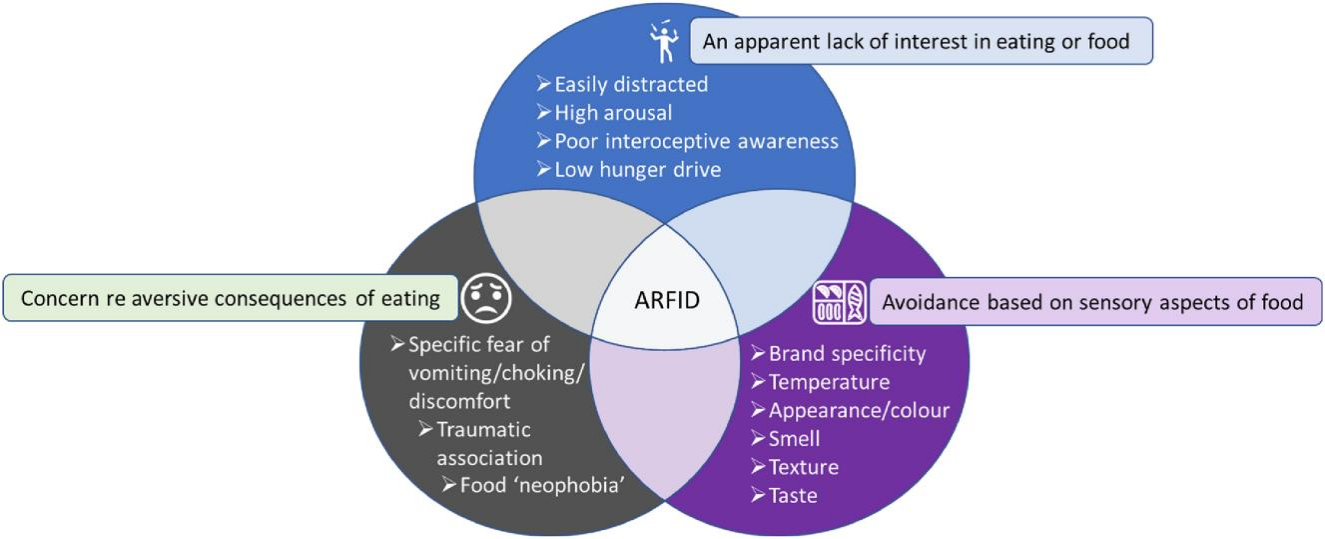
The three most commonly observed drivers of ARFID that are not mutually exclusive. People with ARFID can have one or more simultaneously.

The DSM‐5 definition of ARFID features three core drivers of avoidant or restricted eating behaviour: (1) avoidance based on sensory characteristics of food, (2) concern about aversive consequences related to eating, (3) an apparent lack of interest in eating or food (APA, 2013). These three drivers are not mutually exclusive and individuals with ARFID can experience any combination of the three simultaneously (see Figure 1; Norris et al., 2018: Reilly et al., 2019; Zickgraf, Lane‐Loney, et al., 2019; Zickgraf, Murray, et al., 2019). When avoidant/restrictive eating behaviours persist, physical and psychosocial impairment can result in the form of low weight or weight loss, high weight or excessive weight gain, faltering growth, compromised nutrition, reliance on nutritional supplementation, enteral feeding interventions, significant distress, social avoidance stemming from anxiety around eating and food, and other forms of interference with day‐to‐day functioning. These consequences of the disturbed eating behaviour can vary considerably between individuals meeting diagnostic threshold, in terms of severity, level of risk, and domains of impact. This combination of variable drivers and consequences of the avoidant/restrictive eating behaviour contributes to reference to ‘ARFID profiles’ rather than the condition being understood as having a uniform presentation.

Current consensus‐based guidance recommends that clinical care for ARFID involves assessment and support from a multidisciplinary team (MDT) consisting of mental and physical health care professionals, the latter including medical practitioners and dietitians (Eddy et al., 2019; Norris et al., 2021). MDTs may benefit from access to input by other specialist clinicians such as occupational therapists, speech and language therapists, or medical specialists (e.g. gastroenterologists), to improve diagnostic accuracy, treatment planning and improve overall patient outcomes (Sharp et al., 2017). An MDT approach is also indicated due to identified high rates of co‐occurring mental and physical health conditions as well as neurodiversity in those receiving an ARFID diagnosis. For example, ARFID may be diagnosed alongside depression, anxiety, and obsessive‐compulsive disorder (OCD) if the eating disturbance meets diagnostic threshold and warrants clinical attention in its own right. High rates of co‐occurring physical conditions have also been identified, for example, ARFID may develop in the context of gastroenterological conditions or significant food allergies (Fisher et al., 2014). ARFID may also be diagnosed in the context of neurodevelopmental conditions such as autism spectrum disorder (ASD), attention deficit hyperactivity disorder (ADHD), and intellectual/learning disabilities (I/LD's), where the eating disturbance is directly associated with physical and/or psychosocial impairment and warrants treatment in its own right.

Since ARFID was introduced as a diagnosis, there has been a noticeable increase in clinical and research interest. Scientific publications focussing on ARFID are increasing in number covering aspects of its development, onset, detection, and prevalence as well as optimal approaches to clinical care. However, the body of evidence overall remains limited, with many highlighting the need for further research (e.g. Hay, 2020). This situation can make the delivery of evidence‐based practice challenging.

## AIMS AND METHOD

The main aim of this paper is to present a narrative review of current evidence on ARFID relating to four key areas of relevance to clinical practice: (1) screening and prevalence, (2) assessment and characterisation, (3) treatment interventions and (4) service delivery. An overview and critical evaluation of the literature is presented for each of these four areas. We hope to support clinicians through synthesising and discussing key implications of current findings for day‐to‐day practice, promoting evidence‐informed approaches where the evidence‐base remains limited. We highlight opportunities to build on the evidence required to further inform practice and make recommendations for future research and development initiatives aimed at facilitating optimal service planning, enhanced patient‐centred care, and ultimately improved longer‐term outcomes for individuals with ARFID.

A non‐systematic narrative review methodology was adopted. To identify relevant publications, the search terms “ARFID” and “avoidant restrictive food intake disorder” were entered into PubMed and Google Scholar, two freely available online databases, accessible to clinicians. A total of 4810 records were initially identified through Google Scholar and 472 records were identified through PubMed. Following removal of records based on duplication, irrelevant titles or abstracts, and irrelevant content based on the aims of this review, a total of 74 records was included. This included 27 case studies or series, 22 research reports, 24 review articles, and one meta‐analysis. Citations of interest were also made throughout this review using the references from each of the records included. A narrative synthesis of the evidence is presented, and proposed practice implications derived across the four areas of interest. Gaps in the evidence base were identified to support recommendations for future research aiming to guide clinical practice and enhance patient‐centred care.

## RESULTS

### Screening and prevalence

Accurate epidemiological knowledge is essential for planning appropriate health care provision. By understanding the onset, presence, and distribution of ARFID across general and clinical populations, higher risk groups can be detected (e.g., those who possess vulnerable demographic characteristics or those with specific co‐occurring conditions) and patient care and clinical research priorities identified accordingly (Bryant‐Waugh, 2019). At present, however, robust epidemiological studies on ARFID remain absent.

In their recent review, Dinkler and Bryant‐Waugh (2021) identified nine studies exploring ARFID prevalence rates in general population samples, with authors reporting point prevalence estimates ranging from 0.3% to 15.5% (Chen et al., 2020; Chua et al., 2021; Dinkler et al., 2022; Fitzsimmons‐Craft et al., 2019; Gonçalves et al., 2019; Hay et al., 2017; Hilbert et al., 2021; Kurz et al., 2015; Schmidt et al., 2018). The samples differed in relation to sampling methods, age range, geographical location, and screening approach, making it difficult to arrive at generalisable estimates.

Prevalence estimates across clinical populations are similarly difficult to interpret. The majority of prevalence studies from clinical settings to date have focussed on children and adolescents seen in specialised eating disorder settings or feeding clinics in the USA and Canada (Bryant‐Waugh et al., 2021). In addition, many studies targeting clinical populations have been reliant on retrospective chart studies involving data recorded prior to the inclusion of ARFID in the DSM‐5. Researchers therefore estimated the percentage of patients likely to have received an ARFID diagnosis had one existed at the time, a process likely to compromise reliability of findings. Studies across clinical settings reveal estimated prevalence rates of anywhere between 5% and 32% in these settings (Cooney et al., 2018; Fisher et al., 2014; Forman et al., 2014; Krom et al., 2019; Nicely et al., 2014; Norris et al., 2014; Ornstein et al., 2013; Williams et al., 2015). In other clinical population samples, ARFID prevalence rates have been estimated at 1.5%–8% in paediatric gastroenterology (Eddy et al., 2015; Murray et al., 2022) 6.3% in adult neuro‐gastroenterology (Murray et al., 2020), 0.9% across a general paediatric inpatient sample (Schöffel et al., 2021), and 3.7% in paediatric gynaecology (Goldberg et al., 2020). Recent data from a large autism cohort provided an estimated prevalence rate of 21%, suggesting a high level of risk and co‐occurrence with neurodiversity (Tanner Koomar et al., 2021).

Existing prevalence literature is limited in relation to information about sex and ethnicity, however, available general population studies suggest relatively equal male to female ratios (Dinkler & Bryant‐Waugh, 2021). In addition, recent research exploring the characteristics of ARFID in a child and adolescent clinical sample (*N* = 261) found a similarly equal sex ratio as well as similarities across the sexes in terms of the three core drivers of ARFID (sensory based avoidance, lack of interest, a fear of aversive consequences; Watts et al., 2023). There remain insufficient data to characterise ARFID across different social and ethnic groups, further highlighting the need for more epidemiological studies to identify whether specific groups may be at higher risk.

As ARFID is a relatively new diagnosis, screening instruments have only recently been developed with, to date, no one measure having been identified as having greater utility than others (Bryant‐Waugh, 2019). Currently available screening measures for ARFID tap different aspects of presentations, with limited validation data yet available (Dinkler & Bryant‐Waugh, 2021). Some, such as the Nine‐Item ARFID screen (NAIS; Zickgraf & Ellis, 2018), focus on the core drivers of restricted eating behaviour (i.e., concern about aversive consequences, sensory based avoidance, low interest in food). Others, such as the ARFID‐Brief Screener (ARFID‐BS) (Dinkler et al., 2022) and the Short ARFID Screen (SAS; Bryant‐Waugh et al., 2021; Dinkler et al., 2022) focus predominantly on the clinical implications of ARFID (i.e., nutritional deficiencies, psychosocial impairment, faltering growth and low weight, in line with diagnostic requirements; Dinkler et al., 2022). Additionally, some screening instruments are ARFID specific (NAIS, ARFID‐BS, SAS), whereas others include other eating disorders, such as the Eating Disorders in Youth‐Questionnaire (EDY‐Q; Hilbert & van Dyck, 2016) and the Stanford‐Washington University Eating Disorder Screen (SWED; Graham et al., 2019). Furthermore, some measures are self‐report, some are parent/carer reported, some are clinically administered, and some are currently validated for use in community populations but not in clinical settings. The disparate use of screening measures across studies likely contributes to the differences observed in prevalence estimates, driven in part by inconsistent sensitivity and specificity capacities.

Differing health care systems and levels of knowledge and awareness of ARFID may also impact reported rates. The latter may be of relevance for surveillance studies which rely on professionals reporting identified cases. It is widely acknowledged that current recognition of ARFID presentations and knowledge about the correct application of diagnostic criteria remains limited amongst health care practitioners, many of whom lack experience or confidence in distinguishing ARFID either from normative “picky eating” (Zickgraf, Lane‐Loney, et al., 2019), which can result in over‐identification, or other eating disorders (Coelho et al., 2021; Magel et al., 2021). It may not occur to practitioners to administer screening measures when symptoms of ARFID are observed but not recognised as such. Prevalence rates may therefore be variably over‐ or under reported which currently presents a challenge in understanding data obtained.

Some of the uncertainty surrounding the detection of ARFID may be partly due to how ARFID is defined, as inclusion criteria are open to a degree of interpretation. Diagnostic classifications are primarily intended for clinicians with an element of clinical judgement expected in the conferment of a diagnosis. Consequently, current criteria do not include a clear definition for weight and nutritional symptoms (Zickgraf et al., 2019) or psychosocial impairment. Furthermore, there has been some confusion among clinicians whether experiencing psychosocial impairment alone is enough to diagnose ARFID, or whether individuals must also experience one of the other criteria more closely associated with unmet energy or nutritional needs (Becker et al., 2019; Coelho et al., 2021; Eddy et al., 2019). However, recently updated DSM criteria, as well as the ICD‐11 criteria now make clear that impact of eating difficulties can affect physical health *or* result in psychosocial impairment.

Epidemiological findings therefore vary widely and are limited in terms of number, sample characteristics and size, setting, and geographical region. Most report point prevalence rates rather than lifetime prevalence and screening methodology varies considerably in its robustness and reliability. Little is known about incidence. Despite this, some consistent impressions emerge. Due to people with ARFID presenting to a range of clinical settings, the ongoing differences among health care professionals surrounding knowledge and awareness of ARFID needs to be actively addressed. The detection of possible ARFID cases can, and should, be made by all health care professionals from primary through to tertiary care, and not limited to a particular sub‐group of specialist practitioners (Eddy et al., 2019). Ongoing awareness raising and training on the current conceptualisation of ARFID remains strongly indicated.

### Assessment and characterisation of ARFID

A systematic and standardised approach to the assessment of ARFID is important to characterise the presenting eating difficulty and for objective identification of the key drivers and core contributing and maintaining factors of the avoidant/restrictive eating behaviours. Additionally, a more accurate and informed evaluation of associated nutritional, physical and/or psychological risk is enabled. Co‐occurring mental and physical health conditions and potential differential diagnoses can also be collectively considered and incorporated into diagnostic decision making. Standardised measures at assessment are important not only to guide clinical judgement in the formulation of effective treatment plans, but also enable clinicians to track progress throughout treatment (Bourne et al., 2020). Further, gathering assessment data in a systematic fashion allows us to work towards better characterisation of ARFID, where typical ARFID profiles can be identified and potentially matched to specific treatments (Bryant‐Waugh et al., 2021) thereby improving future clinical management.

Evidence suggests that a significant portion of people with ARFID experience co‐occurring neurodevelopmental diagnoses and mental health conditions including mood and anxiety disorders (Norris et al., 2018, 2021; Okereke, 2018; Zickgraf et al., 2019), ADHD and OCD (Fisher et al., 2014; Nicely et al., 2014; Reilly et al., 2019), autism (Bourne et al., 2022; Farag et al., 2021; Mayes & Zickgraf, 2019), and Internet gaming disorder (Hadwiger et al., 2019). It is therefore important that clinicians utilise the assessment process to better understand the presenting difficulties in the context of other potential co‐occurring conditions.

To develop a broader contextual understanding of the presenting difficulties, ARFID specific measures such as PARDI‐AR‐Q (Pica, ARFID, Rumination Disorder Interview ARFID Questionnaire; Bryant‐Waugh et al., 2021) can be used in combination with short behavioural and developmental screening tools such as the SDQ (Strengths and Difficulties Questionnaire; Goodman, 2001) and the AQ‐10 (The Autism‐spectrum Quotient; Baron‐Cohen et al., 2001). Additionally, measures assessing anxiety and mood status such as the RCADS (Revised Children's Anxiety and Depression Scale; Chorpita et al., 2000) and HoNOSCA (Health of the Nation Outcome Scales, Child and Adolescent mental health; Gowers et al., 1999) can be helpful in determining potential psychological comorbidities and markers for psychological risk. To contextualise the presenting difficulties further, semi‐structured clinical diagnostic interviews can be administered such as the PARDI (Pica, ARFID, Rumination Disorder Interview; Bryant‐Waugh et al., 2019), the SCID‐5 (Structured Clinical Interview for DSM‐5; First et al., 2015), and the EDE‐ARFID (Eating Disorder Examination – ARFID module; Schmidt et al., 2019).

A systematic approach to dietetic evaluation at assessment is important to determine whether people with ARFID have become nutritionally compromised due to their restricted eating. A recent study using a 3‐day food diary compared dietary intake between children with ARFID versus a control group (Schmidt et al., 2021). Results demonstrated that those with ARFID showed reduced variety in their dietary repertoire, significantly lower calorific intake compared to controls, and achieved only 20%–30% of their recommended micro and macro nutrients. Further evidence has shown that a high percentage of children with ARFID were deficient in vitamin D, vitamin E and calcium, in‐addition to having inadequate fibre intake (Sharp et al., 2018). Other deficiencies and impairments identified in the ARFID literature include scurvy, vitamin A, thiamin and vitamin B‐12 (Yule et al., 2021). A food diary which includes records of food and fluid intake can therefore provide a structured method to support dietetic analysis of the range and amount of food and liquid consumed to assess nutritional and health risk.

In addition to implementing structured approaches to the psychological and dietetic components of the assessment process, routine physical examinations allow for any acute or chronic health issues to be identified. Individuals should have their weight and height and, where appropriate, growth trajectories assessed to ascertain whether there is evidence of faltering growth, low weight, or risks of obesity which are commonly observed across people with ARFID (Kerem et al., 2022). It is also important to ascertain whether there is evidence of pubertal delay, and if so, further medical investigations may be indicated (Bryant & Higgins., 2020). Evidence suggests that people with ARFID often present with one or more physical symptoms including abdominal pain, swallowing difficulties, early satiety, or vomiting (Cooney et al., 2018; Fisher et al., 2014; Norris et al., 2014). Consideration of differential diagnoses of medical conditions is important as such symptoms could be related to alternative explanatory conditions. A thorough physical assessment can identify important underlying disorders that may impact eating behaviours including Crohn's disease, ulcerative colitis, and irritable bowel syndrome (Yelencich et al., 2022). Assessing bowel health is also important to determine the presence of constipation which may contribute to, or be a result of, restricted eating or poor fluid intake (Harris et al., 2021). Once all necessary information has been gathered, multi‐disciplinary consideration of collective evidence from a dietetic, medical, and psychological perspective is required before deciding whether the individual meets diagnostic threshold for ARFID.

A recent scoping review of the diagnostic validity of ARFID (Strand et al., 2019) draws attention to some of the challenges health care practitioners experience in diagnostic decision making. Both DSM‐5 and ICD‐11 clearly define the demarcation of ARFID from other eating disorders such as AN or BN, while distinguishing ARFID as a primary disorder over other co‐occurring conditions can elicit diagnostic uncertainty. The DSM‐5 clearly states that the eating disturbance in ARFID should not be entirely attributable to a concurrent medical condition or better explained by another mental disorder. However, the DSM‐5 also states that when the observed eating disturbance does occur in the context of another condition, diagnostic threshold for ARFID may be met when the severity of eating difficulties exceeds that routinely associated with that condition, or warrants additional clinical attention in its own right. For example, not all people with anxiety or autism present with ARFID behaviours, however, when ARFID behaviours are identified to the extent that there is nutritional, physical, and/or psychosocial impairment as a direct result of the eating disturbance, then an ARFID diagnosis would be warranted in the context of the co‐occurring disorder.

In addition, ARFID encompasses a heterogenous set of presenting symptoms, driven by a range of factors that may or may not be related and resulting in variable levels of risk and impairment across a range of domains (i.e. physical, nutritional, psychosocial). As the diagnosis alone does not allow differentiation of this variability, clinicians may face some uncertainty when formulating appropriate targets for clinical management. Characterising the presenting difficulty via a comprehensive assessment process is therefore essential for the development of appropriately tailored and targeted care plans.

### Treatment of ARFID

The adoption of a holistic and formulation‐led approach to treatment planning for ARFID is important for ensuring interventions correspond to the diverse nature of the condition. Consideration is required for the neurodevelopmental profile of the individual, their developmental stage, key contributing and maintaining factors, main drivers, barriers to change, degree of risk, level of impact and the specific goals as outlined by the individual and their family (Bryant‐Waugh et al., 2021; Bryant‐Waugh & Higgins, 2020; Golden et al., 2015). Well‐established standardised treatment protocols for ARFID are yet to be established (Datta et al., 2022) but from what we know so far, it is broadly agreed that treatment options should be multi‐modal in nature (Norris et al., 2021). Prioritisation of the improvement of impaired nutritional status and compromised weight may be required in the first instance, with a psycho‐behavioural intervention required as the main treatment to address the core disturbance in eating behaviour (Hay, 2020).

Positive change in relation to increased body weight has been observed across several studies when cognitive behavioural therapy (CBT), family‐based interventions, exposure therapy, and pharmacological interventions were implemented (Dalle Grave & Sapuppo, 2020). It is notable, however, that weight change is only one of several variables for consideration when measuring treatment outcomes, as improved nutritional status, reduction in psychosocial impairment, and improved physical well‐being also hold significance and may represent a greater priority for any one person (Bryant‐Waugh et al., 2021). Where goals are agreed at the start of treatment, the expectations of the individual and their family can be managed and realistic outcomes for the individual can be openly discussed.

### Psycho‐behavioural interventions

Family‐based and parenting interventions are regularly adopted psycho‐behavioural approaches used in treatment for children and young people with ARFID, typically aiming to support the family to manage meal‐times effectively, decrease food‐related conflict, and for the child/young person to habituate to novel foods or increased intake through repeated exposure (Lesser et al., 2017; Lock, Robinson, et al., 2019; Loeb et al., 2015; Nitsch et al., 2021; Spettigue et al., 2018). SPACE‐ARFID, a parent‐centred intervention for childhood anxiety adapted for ARFID focussing on minimising parental accommodation and increasing food‐related flexibility, has demonstrated promising clinical outcomes (Shimshoni et al., 2020). A range of adapted CBT interventions have also shown promise, and appear particularly beneficial where fear‐related avoidance, emotion regulation difficulties, and general anxiety relating to food are playing a key role in the eating disturbance (Aloi et al., 2018; Bryant‐Waugh et al., 2021; Fischer et al., 2015; Görmez et al., 2018; King et al., 2015; Zimmerman & Fisher, 2017). A CBT intervention adapted for ARFID (CBT‐AR) has recently been developed for people aged 10‐years and over, focussed on supporting individuals with a range of ARFID profiles to increase their quantity of preferred foods before increasing their variety (Brigham et al., 2018). Efficacy data on this open trial are not yet available, however initial results demonstrate promising outcomes (Brigham et al., 2018). In addition to the above, further treatment options include enhanced psychoeducation, group intervention, and core behavioural therapy (Bryant‐Waugh & Higgins, 2020; Bryant‐Waugh et al., 2021). At present no single psycho‐behavioural treatment modality emerges as superior to any other, but family‐based, behavioural, and CBT‐based approaches appear to show greatest promise overall.

### Dietetic interventions

The nature of dietetic intervention depends on which of the core drivers of ARFID are at play, as well as the degree of nutritional risk. For individuals who are experiencing sensory based food avoidance and/or a fear of aversive consequences, preferred foods should predominantly be offered to sustain their calorific intake, while at the same time gradually exposing the individual to very small amounts of previously rejected/feared foods (Coglan & Otasowie, 2019). For individuals who have a low interest in food or poor introspective awareness, recommendations include the implementation of structured routines to ensure mealtimes and behavioural expectations around eating are consistent, the utilisation of behavioural rewards when food is eaten at the table, inclusion of the individual in food related tasks such as weekly shops and food preparation, and increasing portion sizes to maximise calorific intake (Mammel & Ornstein, 2017). Where nutritional risk is a concern, supplements are advised. Supplements can take the form of over‐the‐counter daily multivitamins, or where nutritional deficiencies are present, prescription supplementation may be warranted. Individuals who are at acute nutritional risk may require onward referral for a more intensive level of care, with some requiring a period of enteral feeding.

### Medical and pharmacological interventions

Medical interventions may be required to manage some of the consequences of the eating disturbance which can contribute to maintaining eating difficulties, for example, as in medical management of constipation. Research into the use of medications as an adjunct to psycho‐behavioural therapy is increasing, with initial evidence showing promising results for the use of cyproheptadine as an appetite stimulant (Harrison et al., 2019), D‐cycloserine for reducing aversions to food and improving responses to behavioural therapy (Sharp, Volkert, et al., 2017), and olanzapine for reducing cognitive rigidity (Brewerton & D'Agostino, 2017). Additionally, mirtazapine, fluoxetine, buspirone, and lorazepam have been reported to mitigate the influence of comorbid anxiety, food specific anxiety, or low mood on reduced appetite or avoidant eating behaviours (Gray et al., 2018; Kardas et al., 2014; Norris et al., 2016; Spettigue et al., 2018). Randomised placebo‐controlled trials and larger scale efficacy studies are required before such findings can be considered in terms of clinical recommendations. At present, as with most eating disorders, it seems unlikely that medication will emerge as first line treatment of choice.

### Service delivery

Early intervention is widely regarded as an important factor in improving outcomes for people with eating disorders, and has also been identified as important in young people with ARFID (Kurotori et al., 2019). In England, health care policy sets out that children and young people experiencing eating disorders, including ARFID, should be able to access services easily and receive appropriate input quickly, without barriers to referral and with treatment commencing within a maximum of 4 weeks (NHS, 2019; National Collaborating Centre for Mental Health, 2015). This policy was introduced as a service transformation programme with linked government funding, in part informed by the evidence for early intervention. In their recent overview of the implementation of this policy change, Eisler and colleagues comment on the need for ongoing training in the identification, management and treatment of ARFID to ensure that the principles of ease of access and timely intervention can be meaningfully applied (Eisler et al., 2022). Onward referral to teams or professionals that cannot provide the recommended multidisciplinary input can also delay appropriate treatment for care seeking individuals (Bryant‐Waugh et al., 2021; Coelho et al., 2021) making the development of clear referral pathways important.

Current evidence‐based guidance is that the majority of people with eating disorders including ARFID should receive care in an outpatient or community setting (Hay, 2020). Where generalised anxiety, low mood, or other psychiatric comorbidities are observed, recommendations have been made that additional help from appropriate service providers may be required (Ridgeway & McNicholas, 2021). The use of routine outcome measures has been recommended in outpatient and community settings to monitor progress, treatment response and level of risk (Bryant‐Waugh & Higgins, 2020). Where there is limited response or increased risk after five to six treatment sessions, consideration of adaptations to chosen interventions or the use of alternative treatment strategies is advised (Bryant‐Waugh et al., 2021). Where the level of risk becomes high despite tailored adaptations to care, and where outpatient or community settings feel they can no longer adequately support the individual, a step‐up approach to intervention may be indicated (Coglan & Otasowie, 2019). In such instances, an onwards referral to specialist day programmes or inpatient admission may be required (Hay, 2020).

Reported management of ARFID in specialist day services (Nicely et al., 2014; Sharp et al., 2016) and inpatient settings (Strandjord et al., 2015) varies, with some providers adopting similar treatment methods in low weight ARFID as in AN (Guss et al., 2018). Such methods might include a structured refeeding programme, with or without enteral nutrition, in conjunction with psycho‐behavioural intervention (Hay, 2020). Despite typically presenting with less weight loss than AN patients, some studies have shown that people with ARFID are more likely to rely on enteral nutrition compared to those with AN (Peebles et al., 2017) and are hospitalised for longer (Strandjord et al., 2015). Treatment goals that require smaller incremental increases in calorific intake for ARFID admissions compared to those with AN may contribute to longer hospitalisations (Strandjord et al., 2015). Additionally, people with ARFID may depend on enteral nutrition for longer due to initially presenting at, or being referred to, gastroenterology or other medical settings prior to being diagnosed with ARFID (Brigham et al., 2018). An important treatment goal for some people with ARFID can be to reduce reliance on enteral nutrition and increase oral intake, which can occur both in day programs (Williams et al., 2007) and inpatient settings (Brown et al., 2014; Silverman et al., 2013).

Service delivery models should be set up to accommodate neurodivergent profiles, given the evidence suggesting that a high proportion of individuals within the autistic population experience symptoms of ARFID (Bourne et al., 2022). Similar to neurotypical individuals, those with autism have been reported to respond positively to treatment for avoidant/restricted eating when interventions are conducted by multidisciplinary teams (Keen, 2008; Laud et al., 2009). While each of the three core drivers of avoidant/restricted eating have been found to contribute to ARFID symptoms within the autistic population, reported avoidance of food based on sensory sensitivities has so far featured most in the literature, with concern about aversive consequences of eating being the second most reported driver (Bourne et al., 2022).

Among others, treatments such as stimulus fading (Roth et al., 2010), repeated taste exposure and escape prevention (Paul et al., 2007), bite size evaluation (Sharp & Jaquess, 2009), and sensory integration therapy (Seiverling et al., 2018) have all shown promising success in treating autistic individuals with ARFID. However, like much of the evidence base for ARFID, studies reporting treatment with autistic individuals consist predominantly of single case and case series reports. Consideration of other aspects of neurodiversity will also usefully be factored into service provision, for example, those with ARFID in the context of ADHD may experience restricted eating that is further exacerbated by prescribed stimulant medication (Pennell et al., 2016), and those with intellectual disabilities may present with additional medical complexities and communication impairments (Stone‐Heaberlin et al., 2020), each requiring appropriately adapted treatments.

We know from the prevalence literature and mapping surveys that children and young people with avoidant/restrictive eating behaviours present to a range of health care settings, and considering that many health care teams are not currently set up to provide the level of care required, a well‐defined referral and care pathway needs to be implemented. There may be a requirement for clinicians to work together across services and move towards a joined‐up network of care rather than one standalone service covering all aspects of the presenting difficulties. For example, where people with ARFID present with eating disturbances that co‐occur with other disorders, clinicians from different professional disciplines and settings may be required to work together to provide integrated care appropriate to the individual's needs, covering both physical and mental health aspects of their presentation.

### Future directions

Due to limitations in existing research across all four areas explored, we include here recommendations for ongoing and future attention.

A greater number of well‐designed incidence and prevalence studies based on well‐validated screening and assessment measures are needed across both clinical and non‐clinical settings. These will ideally cover a range of countries and regions, differing health care systems and varying cultural practices. To date, much of the prevalence literature has focussed on children and young people, with a notable lack of research in adult populations, and little by way of data about incidence overall. Peak age of onset and of clinical presentation and the identification of vulnerable groups and clinical populations with higher risk, will be essential to inform future service planning and resource allocation. To further validate available ARFID screening measures, two stage studies are indicated across both community and clinical populations. Such studies might include in depth clinical assessment of sub‐samples of high and low scorers to determine caseness, with sensitivity and specificity analyses of screening measures.

Due to the heterogeneous nature of ARFID, it will be helpful to ensure clinicians are supported to engage in ongoing routine administration of standardised measures across a range of already identified variables throughout assessment and treatment. This will allow the systematic capture of data on clinical presentations and response to treatment interventions. Variables of interest include aspects of ARFID presentation (including drivers, and domains of physical and psychosocial impairment) as well as the presence of neurodiversity and co‐occurring mental and physical health conditions. Such data would support improved interpretation of the findings of treatment trials and help to indicate whether specific treatments might be better matched to certain clinical presentation profiles. While a simple standardised treatment protocol for ARFID appears unlikely, systematically gathered information could in this way help practitioners work towards more evidence‐informed care.

Psychological interventions are currently recommended as the main treatment approach for ARFID, yet robust randomised controlled trial (RCT) data remain absent. A few trials are now underway, but more are needed, with sample sizes allowing for sufficient power and sample characteristics representative of the broad population of people presenting with ARFID. Adequately sized, randomised placebo‐controlled medication trials are needed to determine whether any medications can be reliably recommended specifically for the treatment of ARFID. The impact on the eating disturbance of the use of medication to treat co‐occurring conditions also requires formal evaluation to guide and support clinicians to deliver optimal evidence‐based practice.

Given the recommendation for multi‐disciplinary, multi‐modal treatment, we propose that studies include a range of outcome variables when evaluating the success of treatment interventions; alongside physical parameters such as weight, growth, BMI, these might include improvements to nutritional profile, evidence of an increased range of foods regularly consumed, reduction of mealtime stress, decreased dependency on oral or enteral supplementation, reduction in distress, improvements in day‐to‐day functional impairment and whether the individual and family members consider that they have made progress their own personal goals. For treatments that demonstrate successful outcomes based on rigorous RCT methodology, delivery in routine clinical settings will need to be evaluated and, where appropriate, guidance refined into manualised versions to facilitate effective adoption into clinical practice.

There is much scope to design and evaluate different models of service delivery. While the core elements of clinically informed approaches to the assessment and treatment of ARFID can be found in the literature, there remains little on how services might best be configured. For example, it may prove more beneficial and practical to proceed with networks of care providers across different clinical settings contributing to a local, shared ARFID care pathway rather than seeking to establish stand‐alone specialist ARFID services. Hub and spoke, integrated care and stepped‐care models all lend themselves to formal evaluation. The main aim of such studies would be to provide service planners and practitioners with evidence to guide local service development and configuration, and to inform effective referral and care pathways delivering scalable, evidence‐based, consistent patient care.

## CONCLUSION

Despite its relatively recent introduction as a formal diagnosis, ARFID is not new, with clinical presentations included in this category seen by a range of health care professionals and services. Formal classification of ARFID as one of the treatable feeding and eating disorders in the nosology of mental disorders has stimulated a welcome increase in clinical and research interest, which is overdue given its association with significant physical and psychosocial impairment. The evidence considered in this narrative review demonstrates that while good progress is being made in terms of our understanding and conceptualisation of ARFID, there is much that remains unknown. This situation contributes to ongoing confusion and uncertainty, with some clinical professionals feeling under‐confident and others arguably over‐confident given the current state of knowledge. Nevertheless, some clear pointers emerge from the literature as set out in this review. Continued impetus to raise awareness, improve detection, invest in research and training, support the development of appropriate care pathways, and establish networks of professionals and clinical services, will all be important to support the establishment of well‐informed and effective health care provision. ARFID remains a focus of interest with the body of research literature growing, allowing the much‐needed aim of improving evidence‐based practice and patient‐centred care to continue to be realised.

## AUTHOR CONTRIBUTIONS

**Tanith Archibald**: Conceptualization; Formal analysis; Investigation; Methodology; Writing – original draft. **Rachel Bryant‐Waugh**: Conceptualization; Formal analysis; Methodology; Writing – original draft; Writing – review & editing.

## CONFLICT OF INTEREST STATEMENT

The authors declare no conflicts of interest.

## ETHICAL CONSIDERATIONS

Not applicable for this research review article.

## Data Availability

Data sharing is not applicable to this article as no new data were created or analysed in this study.

## References

[jcv212160-bib-0001] Aloi, M. , Sinopoli, F. , & Segura‐Garcia, C. (2018). A case report of an adult male patient with avoidant/restrictive food intake disorder treated with CBT. Psychiatria Danubina, 30(3), 370–373. 10.24869/psyd.2018.370 30267531

[jcv212160-bib-0002] American Psychiatric Association . (2013). Diagnostic and statistical manual of mental disorders (5th ed.). American Psychiatric Association.

[jcv212160-bib-0003] Baron‐Cohen, S. , Wheelwright, S. , Skinner, R. , Martin, J. , & Clubley, E. (2001). The autism‐spectrum quotient (AQ): Evidence from asperger syndrome/high‐functioning autism, males and females, scientists and mathematicians. Journal of Autism and Developmental Disorders, 31(1), 5–17. 10.1023/a:1005653411471 11439754

[jcv212160-bib-0004] Becker, K. R. , Keshishian, A. C. , Liebman, R. E. , Coniglio, K. A. , Wang, S. B. , Franko, D. L. , Eddy, K. T. , & Thomas, J. J. (2019). Impact of expanded diagnostic criteria for avoidant/restrictive food intake disorder on clinical comparisons with anorexia nervosa. International Journal of Eating Disorders, 52(3), 230–238. 10.1002/eat.22988 30578644 PMC7191972

[jcv212160-bib-0005] Bourne, L. , Bryant‐Waugh, R. , Cook, J. , & Mandy, W. (2020). Avoidant/restrictive food intake disorder: A systematic scoping review of the current literature. Psychiatry Research, 288, 112961. 10.1016/j.psychres.2020.112961 32283448

[jcv212160-bib-0006] Bourne, L. , Mandy, W. , & Bryant‐Waugh, R. (2022). Avoidant/restrictive food intake disorder and severe food selectivity in children and young people with autism: A scoping review. Developmental Medicine and Child Neurology, 64(6), 691–700. 10.1111/dmcn.15139 35112345

[jcv212160-bib-0007] Brewerton, T. D. , & D'Agostino, M. (2017). Adjunctive use of olanzapine in the treatment of avoidant restrictive food intake disorder in children and adolescents in an eating disorders program. Journal of Child and Adolescent Psychopharmacology, 27(10), 920–922. 10.1089/cap.2017.0133 29068721

[jcv212160-bib-0008] Brigham, K. S. , Manzo, L. D. , Eddy, K. T. , & Thomas, J. J. (2018). Evaluation and treatment of avoidant/restrictive food intake disorder (ARFID) in adolescents. Current Pediatrics Reports, 6(2), 107–113. 10.1007/s40124-018-0162-y 31134139 PMC6534269

[jcv212160-bib-0009] Brown, J. , Kim, C. , Lim, A. , Brown, S. , Desai, H. , Volker, L. , & Katz, M. (2014). Successful gastrostomy tube weaning program using an intensive multidisciplinary team approach. Journal of Pediatric Gastroenterology and Nutrition, 58(6), 743–749. 10.1097/mpg.0000000000000336 24509305

[jcv212160-bib-0010] Bryant‐Waugh, R. (2019). Avoidant/restrictive food intake disorder. Child and Adolescent Psychiatric Clinics, 28(4), 557–565. 10.1016/j.chc.2019.05.004 31443874

[jcv212160-bib-0011] Bryant‐Waugh, R. , & Higgins, C. (2020). Avoidant restrictive food intake disorder in childhood and adolescence: A clinical guide. Routledge.

[jcv212160-bib-0012] Bryant‐Waugh, R. , Loomes, R. , Munuve, A. , & Rhind, C. (2021). Towards an evidence‐based out‐patient care pathway for children and young people with avoidant restrictive food intake disorder. Journal of Behavioral and Cognitive Therapy, 31(1), 15–26. 10.1016/j.jbct.2020.11.001

[jcv212160-bib-0013] Bryant‐Waugh, R. , Micali, N. , Cooke, L. , Lawson, E. A. , Eddy, K. T. , & Thomas, J. J. (2019). Development of the Pica, ARFID, and rumination disorder interview, a multi‐informant, semi‐structured interview of feeding disorders across the lifespan: A pilot study for ages 10–22. International Journal of Eating Disorders, 52(4), 378–387. 10.1002/eat.22958 30312485 PMC6453710

[jcv212160-bib-0014] Chen, Y.‐L. , Chen, W. J. , Lin, K.‐C. , Shen, L.‐J. , & Gau, S. S.‐F. (2020). Prevalence of DSM‐5 mental disorders in a nationally representative sample of children in Taiwan: Methodology and main findings. Epidemiology and Psychiatric Sciences, 29, e15. 10.1017/s2045796018000793 PMC8061245 30696515

[jcv212160-bib-0015] Chorpita, B. F. , Yim, L. , Moffitt, C. , Umemoto, L. A. , & Francis, S. E. (2000). Assessment of symptoms of DSM‐IV anxiety and depression in children: A revised child anxiety and depression scale. Behaviour Research and Therapy, 38(8), 835–855. 10.1016/s0005-7967(99)00130-8 10937431

[jcv212160-bib-0016] Chua, S. N. , Fitzsimmons‐Craft, E. E. , Austin, S. B. , Wilfley, D. E. , & Taylor, C. B. (2021). Estimated prevalence of eating disorders in Singapore. International Journal of Eating Disorders, 54(1), 7–18. 10.1002/eat.23440 33314277 PMC8011933

[jcv212160-bib-0017] Coelho, J. S. , Norris, M. L. , Tsai, S. C. , Wu, Y. J. , & Lam, P. Y. (2021). Health professionals' familiarity and experience with providing clinical care for pediatric avoidant/restrictive food intake disorder. International Journal of Eating Disorders, 54(4), 587–594. 10.1002/eat.23438 33300613

[jcv212160-bib-0018] Coglan, L. , & Otasowie, J. (2019). Avoidant/restrictive food intake disorder: What do we know so far? BJPsych Advances, 25(2), 90–98. 10.1192/bja.2018.48

[jcv212160-bib-0019] Cooney, M. , Lieberman, M. , Guimond, T. , & Katzman, D. K. (2018). Clinical and psychological features of children and adolescents diagnosed with avoidant/restrictive food intake disorder in a pediatric tertiary care eating disorder program: A descriptive study. Journal of Eating Disorders, 6(1), 1–8. 10.1186/s40337-018-0193-3 29736239 PMC5922027

[jcv212160-bib-0020] Dalle Grave, A. , & Sapuppo, W. (2020). Treatment of avoidant/restrictive food intake disorder: A systematic review. Italian Journal of Eating Disorders and Obesity, 4, 13–23. 10.32044/ijedo.2020.04

[jcv212160-bib-0021] Datta, N. , Matheson, B. E. , Citron, K. , Van Wye, E. M. , & Lock, J. D. (2022). Evidence based update on psychosocial treatments for eating disorders in children and adolescents. Journal of Clinical Child and Adolescent Psychology, 52(2), 1–12. 10.1080/15374416.2022.2109650 35950931

[jcv212160-bib-0022] Dinkler, L. , & Bryant‐Waugh, R. (2021). Assessment of avoidant restrictive food intake disorder, pica and rumination disorder: Interview and questionnaire measures. Current Opinion in Psychiatry, 34(6), 532–542. 10.1097/yco.0000000000000736 34402460

[jcv212160-bib-0023] Dinkler, L. , Yasumitsu‐Lovell, K. , Eitoku, M. , Fujieda, M. , Suganuma, N. , Hatakenaka, Y. , Hadjikhani, N. , Bryant‐Waugh, R. , Råstam, M. , & Gillberg, C. (2022). Development of a parent‐reported screening tool for avoidant/restrictive food intake disorder (ARFID): Initial validation and prevalence in 4‐7‐year‐old Japanese children. Appetite, 168, 105735. 10.1016/j.appet.2021.105735 34626753

[jcv212160-bib-0024] Eddy, K. T. , Harshman, S. G. , Becker, K. R. , Bern, E. , Bryant‐Waugh, R. , Hilbert, A. , Katzman, D. K. , Lawson, E. A. , Manzo, L. D. , Menzel, J. , Micali, N. , Ornstein, R. , Sally, S. , Serinsky, S. P. , Sharp, W. , Stubbs, K. , Walsh, B. T. , Zickgraf, H. , Zucker, N. , & Thomas, J. J. (2019). Radcliffe ARFID Workgroup: Toward operationalization of research diagnostic criteria and directions for the field. International Journal of Eating Disorders, 52(4), 361–366. 10.1002/eat.23042 30758864 PMC6485247

[jcv212160-bib-0025] Eddy, K. T. , Thomas, J. J. , Hastings, E. , Edkins, K. , Lamont, E. , Nevins, C. M. , Patterson, R. M. , Murray, H. B. , Bryant‐Waugh, R. , & Becker, A. E. (2015). Prevalence of DSM‐5 avoidant/restrictive food intake disorder in a pediatric gastroenterology healthcare network. International Journal of Eating Disorders, 48(5), 464–470. 10.1002/eat.22350 25142784

[jcv212160-bib-0026] Eisler, I. , Simic, M. , Fonagy, P. , & Bryant‐Waugh, R. (2022). Implementing service transformation for children and adolescents with eating disorders across England: The theory, politics, and pragmatics of large‐scale service reform. Journal of Eating Disorders, 10(1), 146. 10.1186/s40337-022-00665-z 36217209 PMC9549853

[jcv212160-bib-0027] Farag, F. , Sims, A. , Strudwick, K. , Carrasco, J. , Waters, A. , Ford, V. , Hopkins, J. , Whitlingum, G. , Absoud, M. , & Kelly, V. B. (2021). Avoidant/restrictive food intake disorder and autism spectrum disorder: Clinical implications for assessment and management. Developmental Medicine and Child Neurology, 64(2), 176–182. 10.1111/dmcn.14977 34405406

[jcv212160-bib-0028] First, M. B. , Williams, J. B. , Karg, R. S. , & Spitzer, R. L. (2015). Structured clinical interview for DSM‐5—Research version (SCID‐5 for DSM‐5, research version; SCID‐5‐RV) (pp. 1–94). American Psychiatric Association.

[jcv212160-bib-0029] Fischer, A. J. , Luiselli, J. K. , & Dove, M. B. (2015). Effects of clinic and in‐home treatment on consumption and feeding‐associated anxiety in an adolescent with avoidant/restrictive food intake disorder. Clinical Practice in Pediatric Psychology, 3(2), 154–166. 10.1037/cpp0000090

[jcv212160-bib-0030] Fisher, M. M. , Rosen, D. S. , Ornstein, R. M. , Mammel, K. A. , Katzman, D. K. , Rome, E. S. , Callahan, S. T. , Malizio, J. , Kearney, S. , & Walsh, B. T. (2014). Characteristics of avoidant/restrictive food intake disorder in children and adolescents: A “new disorder” in DSM‐5. Journal of Adolescent Health, 55(1), 49–52. 10.1016/j.jadohealth.2013.11.013 24506978

[jcv212160-bib-0031] Fitzsimmons‐Craft, E. E. , Balantekin, K. N. , Graham, A. K. , Smolar, L. , Park, D. , Mysko, C. , Funk, B. , Taylor, C. B. , & Wilfley, D. E. (2019). Results of disseminating an online screen for eating disorders across the US: Reach, respondent characteristics, and unmet treatment need. International Journal of Eating Disorders, 52(6), 721–729. 10.1002/eat.23043 30761560 PMC6555644

[jcv212160-bib-0032] Forman, S. F. , McKenzie, N. , Hehn, R. , Monge, M. C. , Kapphahn, C. J. , Mammel, K. A. , Callahan, S. T. , Sigel, E. J. , Bravender, T. , Romano, M. , Rome, E. S. , Robinson, K. A. , Fisher, M. , Malizio, J. B. , Rosen, D. S. , Hergenroeder, A. C. , Buckelew, S. M. , Jay, M. S. , Lindenbaum, J. , & Woods, E. R. (2014). Predictors of outcome at 1 year in adolescents with DSM‐5 restrictive eating disorders: Report of the national eating disorders quality improvement collaborative. Journal of Adolescent Health, 55(6), 750–756. 10.1016/j.jadohealth.2014.06.014 25200345

[jcv212160-bib-0033] Goldberg, H. R. , Katzman, D. K. , Allen, L. , Martin, S. , Sheehan, C. , Kaiserman, J. , Macdonald, G. , & Kives, S. (2020). The prevalence of children and adolescents at risk for avoidant restrictive food intake disorder in a pediatric and adolescent gynecology clinic. Journal of Pediatric and Adolescent Gynecology, 33(5), 466–469. 10.1016/j.jpag.2020.06.004 32553711

[jcv212160-bib-0034] Golden, N. H. , Katzman, D. K. , Sawyer, S. M. , Ornstein, R. M. , Rome, E. S. , Garber, A. K. , Kohn, M. , & Kreipe, R. E. (2015). Position paper of the society for adolescent health and medicine: Medical management of restrictive eating disorders in adolescents and young adults. Journal of Adolescent Health, 56(1), 121–125. 10.1016/j.jadohealth.2014.10.259 25530605

[jcv212160-bib-0035] Gonçalves, S. , Vieira, A. I. , Machado, B. C. , Costa, R. , Pinheiro, J. , & Conceiçao, E. (2019). Avoidant/restrictive food intake disorder symptoms in children: Associations with child and family variables. Children's Health Care, 48(3), 301–313. 10.1080/02739615.2018.1532796

[jcv212160-bib-0036] Goodman, R. (2001). Psychometric properties of the strengths and difficulties questionnaire. Journal of the American Academy of Child & Adolescent Psychiatry, 40(11), 1337–1345. 10.1097/00004583-200111000-00015 11699809

[jcv212160-bib-0037] Görmez, A. , Kılıç, A. , & Kırpınar, İ. (2018). Avoidant/restrictive food intake disorder: An adult case responding to cognitive behavioral therapy. Clinical Case Studies, 17(6), 443–452. 10.1177/1534650118795286

[jcv212160-bib-0111] Gowers, S. G. , Harrington, R. C. , Whitton, A ., Lelliott, P ., Beevor, A ., Wing, J ., & Jezzard, R . (1999). Brief scale for measuring the outcomes of emotional and behavioural disorders in children: Health of the Nation Outcome Scales for Children and Adolescents (HoNOSCA). The British Journal of Psychiatry, 174(5), 413–416. 10616607 10.1192/bjp.174.5.413

[jcv212160-bib-0039] Graham, A. K. , Trockel, M. , Weisman, H. , Fitzsimmons‐Craft, E. E. , Balantekin, K. N. , Wilfley, D. E. , & Taylor, C. B. (2019). A screening tool for detecting eating disorder risk and diagnostic symptoms among college‐age women. Journal of American College Health, 67(4), 357–366. 10.1080/07448481.2018.1483936 29979922 PMC6320726

[jcv212160-bib-0040] Gray, E. , Chen, T. , Menzel, J. , Schwartz, T. , & Kaye, W. H. (2018). Mirtazapine and weight gain in avoidant and restrictive food intake disorder. Journal of the American Academy of Child & Adolescent Psychiatry, 57(4), 288–289. 10.1016/j.jaac.2018.01.011 29588055

[jcv212160-bib-0041] Guss, C. E. , Richmond, T. K. , & Forman, S. (2018). A survey of physician practices on the inpatient medical stabilization of patients with avoidant/restrictive food intake disorder. Journal of Eating Disorders, 6(1), 22. 10.1186/s40337-018-0212-4 30263118 PMC6157044

[jcv212160-bib-0042] Hadwiger, A. N. , Middleman, A. B. , & Pitt, P. D. (2019). Case series: Gaming vs. eating—Comorbidity of ARFID and IGD. Eating and Weight Disorders‐Studies on Anorexia, Bulimia and Obesity, 24(5), 959–962. 10.1007/s40519-019-00639-2 30788778

[jcv212160-bib-0043] Harris, H. A. , Micali, N. , Moll, H. A. , van Berckelaer‐Onnes, I. , Hillegers, M. , & Jansen, P. W. (2021). The role of food selectivity in the association between child autistic traits and constipation. International Journal of Eating Disorders, 54(6), 981–985. 10.1002/eat.23485 33594728 PMC8248436

[jcv212160-bib-0044] Harrison, M. E. , Norris, M. L. , Robinson, A. , Spettigue, W. , Morrissey, M. , & Isserlin, L. (2019). Use of cyproheptadine to stimulate appetite and body weight gain: A systematic review. Appetite, 137, 62–72. 10.1016/j.appet.2019.02.012 30825493

[jcv212160-bib-0045] Hay, P. (2020). Current approach to eating disorders: A clinical update. Internal Medicine Journal, 50(1), 24–29. 10.1111/imj.14691 31943622 PMC7003934

[jcv212160-bib-0046] Hay, P. , Mitchison, D. , Collado, A. E. L. , González‐Chica, D. A. , Stocks, N. , & Touyz, S. (2017). Burden and health‐related quality of life of eating disorders, including Avoidant/Restrictive Food Intake Disorder (ARFID), in the Australian population. Journal of Eating Disorders, 5(1), 1–10. 10.1186/s40337-017-0149-z 28680630 PMC5494787

[jcv212160-bib-0047] Hilbert, A. , & van Dyck, Z. (2016). Eating disorders in youth‐questionnaire. English version University of Leipzig. http://nbn‐resolving.de/urn:nbn:de:bsz

[jcv212160-bib-0048] Hilbert, A. , Zenger, M. , Eichler, J. , & Brähler, E. (2021). Psychometric evaluation of the Eating Disorders in Youth‐Questionnaire when used in adults: Prevalence estimates for symptoms of avoidant/restrictive food intake disorder and population norms. International Journal of Eating Disorders, 54(3), 399–408. 33283329 10.1002/eat.23424

[jcv212160-bib-0049] Kardas, M. , Cermik, B. B. , Ekmekci, S. , Uzuner, S. , & Gökçe, S. (2014). Lorazepam in the treatment of posttraumatic feeding disorder. Journal of Child and Adolescent Psychopharmacology, 24(5), 296–297. 10.1089/cap.2013.0149 24813692

[jcv212160-bib-0050] Keen, D. V. (2008). Childhood autism, feeding problems and failure to thrive in early infancy. European Child & Adolescent Psychiatry, 17(4), 209–216. 10.1007/s00787-007-0655-7 17876499

[jcv212160-bib-0051] Kerem, L. , Van De Water, A. L. , Kuhnle, M. C. , Harshman, S. , Hauser, K. , Eddy, K. T. , Becker, K. R. , Misra, M. , Micali, N. , Thomas, J. J. , Holsen, L. , & Lawson, E. A. (2022). Neurobiology of avoidant/restrictive food intake disorder in youth with overweight/obesity versus healthy weight. Journal of Clinical Child and Adolescent Psychology, 51(5), 701–714. 10.1080/15374416.2021.1894944 33769133 PMC8464625

[jcv212160-bib-0052] King, L. A. , Urbach, J. R. , & Stewart, K. E. (2015). Illness anxiety and avoidant/restrictive food intake disorder: Cognitive‐behavioral conceptualization and treatment. Eating Behaviors, 19, 106–109. 10.1016/j.eatbeh.2015.05.010 26276708

[jcv212160-bib-0053] Krom, H. , van der Sluijs Veer, L. , van Zundert, S. , Otten, M. A. , Benninga, M. , Haverman, L. , & Kindermann, A. (2019). Health related quality of life of infants and children with avoidant restrictive food intake disorder. International Journal of Eating Disorders, 52(4), 410–418. 10.1002/eat.23037 30734346 PMC6594067

[jcv212160-bib-0054] Kurotori, I. , Shioda, K. , Abe, T. , Kato, R. , Ishikawa, S. , & Suda, S. (2019). An inpatient observational study: Characteristics and outcomes of avoidant/restrictive food intake disorder (ARFID) in children and adolescents in Japan. Neuropsychiatric Disease and Treatment, 15, 3313–3321. 10.2147/ndt.s218354 31819456 PMC6886540

[jcv212160-bib-0055] Kurz, S. , Van Dyck, Z. , Dremmel, D. , Munsch, S. , & Hilbert, A. (2015). Early‐onset restrictive eating disturbances in primary school boys and girls. European Child & Adolescent Psychiatry, 24(7), 779–785. 10.1007/s00787-014-0622-z 25296563 PMC4490181

[jcv212160-bib-0056] Laud, R. B. , Girolami, P. A. , Boscoe, J. H. , & Gulotta, C. S. (2009). Treatment outcomes for severe feeding problems in children with autism spectrum disorder. Behavior Modification, 33(5), 520–536. 10.1177/0145445509346729 19748900

[jcv212160-bib-0057] Lesser, J. , Eckhardt, S. , Ehrenreich‐May, J. , & Le Grange, D. (2017). Integrating family based treatment with the unified protocol for the transdiagnostic treatment of emotional disorders: A novel treatment for avoidant restrictive food intake disorder. Academy of Eating Disorders International Conference.

[jcv212160-bib-0058] Lock, J. , Robinson, A. , Sadeh‐Sharvit, S. , Rosania, K. , Osipov, L. , Kirz, N. , Derenne, J. , & Utzinger, L. (2019). Applying family‐based treatment (FBT) to three clinical presentations of avoidant/restrictive food intake disorder: Similarities and differences from FBT for anorexia nervosa. International Journal of Eating Disorders, 52(4), 439–446. 10.1002/eat.22994 30578635

[jcv212160-bib-0060] Loeb, K. L. , Le Grange, D. , & Lock, J. (2015). Family therapy for adolescent eating and weight disorders: New applications. Routledge.

[jcv212160-bib-0061] Magel, C. , Hewitt, K. , Dimitropoulos, G. , von Ranson, K. , & McMorris, C. (2021). Who is treating ARFID, and how? The need for training for community clinicians. Eating and Weight Disorders‐Studies on Anorexia, Bulimia and Obesity, 26(4), 1279–1280. 10.1007/s40519-020-01007-1 32926345

[jcv212160-bib-0062] Mammel, K. A. , & Ornstein, R. M. (2017). Avoidant/restrictive food intake disorder: A new eating disorder diagnosis in the diagnostic and statistical manual 5. Current Opinion in Pediatrics, 29(4), 407–413. 10.1097/mop.0000000000000507 28537947

[jcv212160-bib-0063] Mayes, S. D. , & Zickgraf, H. (2019). Atypical eating behaviors in children and adolescents with autism, ADHD, other disorders, and typical development. Research in Autism Spectrum Disorders, 64, 76–83. 10.1016/j.rasd.2019.04.002

[jcv212160-bib-0064] Murray, H. B. , Bailey, A. P. , Keshishian, A. C. , Silvernale, C. J. , Staller, K. , Eddy, K. T. , Thomas, J. J. , & Kuo, B. (2020). Prevalence and characteristics of avoidant/restrictive food intake disorder in adult neurogastroenterology patients. Clinical Gastroenterology and Hepatology, 18(9), 1995–2002.e1991. 10.1016/j.cgh.2019.10.030 31669056

[jcv212160-bib-0065] Murray, H. B. , Rao, F. U. , Baker, C. , Silvernale, C. J. , Staller, K. , Harshman, S. G. , Thomas, J. J. , Kuo, B. , & Zar‐Kessler, C. (2022). Prevalence and characteristics of avoidant/restrictive food intake disorder in pediatric neurogastroenterology patients. Journal of Pediatric Gastroenterology and Nutrition, 74(5), 588–592. 10.1097/mpg.0000000000003369 34908014 PMC10126824

[jcv212160-bib-0066] National Collaborating Centre for Mental Health . (2015). Access and waiting time standard for children and young people with an eating disorder. https://www.england.nhs.uk/wp‐content/uploads/2015/07/cyp‐eating‐disorders‐access‐waiting‐time‐standard‐comm‐guid.pd

[jcv212160-bib-0068] NHS England . (2019). The NHS long term plan. https://www.longtermplan.nhs.uk/wp‐content/uploads/2019/08/nhs‐long‐term‐plan‐version‐1.2.pdf

[jcv212160-bib-0069] Nicely, T. A. , Lane‐Loney, S. , Masciulli, E. , Hollenbeak, C. S. , & Ornstein, R. M. (2014). Prevalence and characteristics of avoidant/restrictive food intake disorder in a cohort of young patients in day treatment for eating disorders. Journal of Eating Disorders, 2(1), 1–8. 10.1186/s40337-014-0021-3 25165558 PMC4145233

[jcv212160-bib-0070] Nitsch, A. , Knopf, E. , Manwaring, J. , & Mehler, P. S. (2021). Avoidant/Restrictive Food Intake Disorder (ARFID): Its medical complications and their treatment—An emerging area. Current Pediatrics Reports, 9(2), 21–29. 10.1007/s40124-021-00239-8

[jcv212160-bib-0071] Norris, M. L. , Obeid, N. , Santos, A. , Valois, D. D. , Isserlin, L. , Feder, S. , & Spettigue, W. (2021). Treatment needs and rates of mental health comorbidity in adolescent patients with ARFID [Brief research report]. Frontiers in Psychiatry, 12. 10.3389/fpsyt.2021.680298 PMC8327955 34349680

[jcv212160-bib-0072] Norris, M. L. , Robinson, A. , Obeid, N. , Harrison, M. , Spettigue, W. , & Henderson, K. (2014). Exploring avoidant/restrictive food intake disorder in eating disordered patients: A descriptive study. International Journal of Eating Disorders, 47(5), 495–499. 10.1002/eat.22217 24343807

[jcv212160-bib-0074] Norris, M. L. , Spettigue, W. J. , & Katzman, D. K. (2016). Update on eating disorders: Current perspectives on avoidant/restrictive food intake disorder in children and youth. Neuropsychiatric Disease and Treatment, 12, 213. 10.2147/ndt.s82538 26855577 PMC4725687

[jcv212160-bib-0073] Norris, M. L. , Spettigue, W. , Hammond, N. G. , Katzman, D. K. , Zucker, N. , Yelle, K. , Santos, A. , Gray, M. , & Obeid, N. (2018). Building evidence for the use of descriptive subtypes in youth with avoidant restrictive food intake disorder. International Journal of Eating Disorders, 51(2), 170–173. 10.1002/eat.22814 29215749

[jcv212160-bib-0075] Okereke, N. K. (2018). Buspirone treatment of anxiety in an adolescent female with avoidant/restrictive food intake disorder. Journal of Child and Adolescent Psychopharmacology, 28(6), 425–426. 10.1089/cap.2018.0005 29812956

[jcv212160-bib-0076] Ornstein, R. M. , Rosen, D. S. , Mammel, K. A. , Callahan, S. T. , Forman, S. , Jay, M. S. , Fisher, M. , Rome, E. , & Walsh, B. T. (2013). Distribution of eating disorders in children and adolescents using the proposed DSM‐5 criteria for feeding and eating disorders. Journal of Adolescent Health, 53(2), 303–305. 10.1016/j.jadohealth.2013.03.025 23684215

[jcv212160-bib-0077] Paul, C. , Williams, K. E. , Riegel, K. , & Gibbons, B. (2007). Combining repeated taste exposure and escape prevention: An intervention for the treatment of extreme food selectivity. Appetite, 49(3), 708–711. 10.1016/j.appet.2007.07.012 17920728

[jcv212160-bib-0078] Peebles, R. , Lesser, A. , Park, C. C. , Heckert, K. , Timko, C. A. , Lantzouni, E. , Liebman, R. , & Weaver, L. (2017). Outcomes of an inpatient medical nutritional rehabilitation protocol in children and adolescents with eating disorders. Journal of Eating Disorders, 5(1), 7. 10.1186/s40337-017-0134-6 28265411 PMC5331684

[jcv212160-bib-0079] Pennell, A. , Couturier, J. , Grant, C. , & Johnson, N. (2016). Severe avoidant/restrictive food intake disorder and coexisting stimulant treated attention deficit hyperactivity disorder. International Journal of Eating Disorders, 49(11), 1036–1039. 10.1002/eat.22602 27521251

[jcv212160-bib-0080] Reilly, E. E. , Brown, T. A. , Gray, E. K. , Kaye, W. H. , & Menzel, J. E. (2019). Exploring the cooccurrence of behavioural phenotypes for avoidant/restrictive food intake disorder in a partial hospitalization sample. European Eating Disorders Review, 27(4), 429–435. 10.1002/erv.2670 30868707

[jcv212160-bib-0081] Ridgeway, L. , & McNicholas, F. (2021). Clinical management of avoidant restrictive food intake disorder (ARFID). Irish Medical Journal, 114(4), 331.

[jcv212160-bib-0082] Roth, M. P. , Williams, K. E. , & Paul, C. M. (2010). Treating food and liquid Refusal in an adolescent with Asperger’s disorder. Clinical Case Studies, 9(4), 260–272. 10.1177/1534650110373500

[jcv212160-bib-0083] Schmidt, R. , Hiemisch, A. , Kiess, W. , von Klitzing, K. , Schlensog‐Schuster, F. , & Hilbert, A. (2021). Macro‐and micronutrient intake in children with avoidant/restrictive food intake disorder. Nutrients, 13(2), 400. 10.3390/nu13020400 33513954 PMC7911718

[jcv212160-bib-0084] Schmidt, R. , Kirsten, T. , Hiemisch, A. , Kiess, W. , & Hilbert, A. (2019). Interview‐based assessment of avoidant/restrictive food intake disorder (ARFID): A pilot study evaluating an ARFID module for the eating disorder examination. International Journal of Eating Disorders, 52(4), 388–397. 10.1002/eat.23063 30843618

[jcv212160-bib-0085] Schmidt, R. , Vogel, M. , Hiemisch, A. , Kiess, W. , & Hilbert, A. (2018). Pathological and non‐pathological variants of restrictive eating behaviors in middle childhood: A latent class analysis. Appetite, 127, 257–265. 10.1016/j.appet.2018.04.030 29738782

[jcv212160-bib-0086] Schöffel, H. , Hiemisch, A. , Kiess, W. , Hilbert, A. , & Schmidt, R. (2021). Characteristics of avoidant/restrictive food intake disorder in a general paediatric inpatient sample. European Eating Disorders Review, 29(1), 60–73. 10.1002/erv.2799 33089950

[jcv212160-bib-0087] Seiverling, L. , Anderson, K. , Rogan, C. , Alaimo, C. , Argott, P. , & Panora, J. (2018). A comparison of a behavioral feeding intervention with and without pre‐meal sensory integration therapy. Journal of Autism and Developmental Disorders, 48(10), 3344–3353. 10.1007/s10803-018-3604-z 29744703

[jcv212160-bib-0088] Sharp, W. , Allen, A. , Stubbs, K. , Criado, K. , Sanders, R. , McCracken, C. , Parsons, R. , Scahill, L. , & Gourley, S. (2017). Successful pharmacotherapy for the treatment of severe feeding aversion with mechanistic insights from cross‐species neuronal remodeling. Translational Psychiatry, 7(6), e1157. 10.1038/tp.2017.126 28632204 PMC5537647

[jcv212160-bib-0089] Sharp, W. G. , & Jaquess, D. L. (2009). Bite size and texture assessments to prescribe treatment for severe food selectivity in autism. Behavioral Interventions: Theory & Practice in Residential & Community‐Based Clinical Programs, 24(3), 157–170. 10.1002/bin.282

[jcv212160-bib-0090] Sharp, W. G. , Postorino, V. , McCracken, C. E. , Berry, R. C. , Criado, K. K. , Burrell, T. L. , & Scahill, L. (2018). Dietary intake, nutrient status, and growth parameters in children with autism spectrum disorder and severe food selectivity: An electronic medical record review. Journal of the Academy of Nutrition and Dietetics, 118(10), 1943–1950. 10.1016/j.jand.2018.05.005 30005820

[jcv212160-bib-0091] Sharp, W. G. , Stubbs, K. H. , Adams, H. , Wells, B. M. , Lesack, R. S. , Criado, K. K. , Simon, E. L. , McCracken, C. E. , West, L. L. , & Scahill, L. D. (2016). Intensive, manual‐based intervention for pediatric feeding disorders: Results from a randomized pilot trial. Journal of Pediatric Gastroenterology and Nutrition, 62(4), 658–663. 10.1097/mpg.0000000000001043 26628445

[jcv212160-bib-0092] Sharp, W. G. , Volkert, V. M. , Scahill, L. , McCracken, C. E. , & McElhanon, B. (2017). A systematic review and meta‐analysis of intensive multidisciplinary intervention for pediatric feeding disorders: How standard is the standard of care? The Journal of Pediatrics, 181, 116–124. 10.1016/j.jpeds.2016.10.002 27843007

[jcv212160-bib-0093] Shimshoni, Y. , Silverman, W. K. , & Lebowitz, E. R. (2020). SPACE‐ARFID: A pilot trial of a novel parent‐based treatment for avoidant/restrictive food intake disorder. International Journal of Eating Disorders, 53(10), 1623–1635. 10.1002/eat.23341 33464594

[jcv212160-bib-0094] Silverman, A. H. , Kirby, M. , Clifford, L. M. , Fischer, E. , Berlin, K. S. , Rudolph, C. D. , & Noel, R. J. (2013). Nutritional and psychosocial outcomes of gastrostomy tube–dependent children completing an intensive inpatient behavioral treatment program. Journal of Pediatric Gastroenterology and Nutrition, 57(5), 668–672. 10.1097/MPG.0b013e3182a027a3 23783012

[jcv212160-bib-0095] Spettigue, W. , Norris, M. L. , Santos, A. , & Obeid, N. (2018). Treatment of children and adolescents with avoidant/restrictive food intake disorder: A case series examining the feasibility of family therapy and adjunctive treatments. Journal of Eating Disorders, 6(1), 1–11. 10.1186/s40337-018-0205-3 30123505 PMC6091012

[jcv212160-bib-0096] Stone‐Heaberlin, M. , Merrill, A. , & Fodstad, J. C. (2020). Feeding problems and assessment in individuals with intellectual disability. In Handbook of dual diagnosis (pp. 357–365). Springer.

[jcv212160-bib-0097] Strand, M. , von Hausswolff‐Juhlin, Y. , & Welch, E. (2019). A systematic scoping review of diagnostic validity in avoidant/restrictive food intake disorder. International Journal of Eating Disorders, 52(4), 331–360. 10.1002/eat.22962 30489647

[jcv212160-bib-0098] Strandjord, S. E. , Sieke, E. H. , Richmond, M. , & Rome, E. S. (2015). Avoidant/restrictive food intake disorder: Illness and hospital course in patients hospitalized for nutritional insufficiency. Journal of Adolescent Health, 57(6), 673–678. 10.1016/j.jadohealth.2015.08.003 26422290

[jcv212160-bib-0099] Tanner Koomar, T. R. T. , Pottschmidt, N. R. , Lutter, M. , & Michaelson, J. J. (2021). Estimating the prevalence and genetic risk mechanisms of ARFID in a large autism cohort. Frontiers in Psychiatry, 12. 10.3389/fpsyt.2021.668297 PMC8221394 34177659

[jcv212160-bib-0100] Watts, R. , Archibald, T. , Hembry, P. , Howard, M. , Kelly, C. , Loomes, R. , Markham, L. , Moss, H. , Munuve, A. , Oros A. , Siddall, A. , Rhind, C. , Uddin M. , Ahmad, Z. , Bryant‐Waugh, R. , Hübel, C. (2023). The clinical presentation of avoidant restrictive food intake disorder is largely independent of sex, autism spectrum disorder and anxiety traits [Manuscript submitted for publication]. medRxiv preprint. 10.1101/2023.02.12.23285723 PMC10480549 37680940

[jcv212160-bib-0101] Williams, K. E. , Hendy, H. M. , Field, D. G. , Belousov, Y. , Riegel, K. , & Harclerode, W. (2015). Implications of avoidant/restrictive food intake disorder (ARFID) on children with feeding problems. Children's Health Care, 44(4), 307–321. 10.1080/02739615.2014.921789

[jcv212160-bib-0102] Williams, K. E. , Riegel, K. , Gibbons, B. , & Field, D. G. (2007). Intensive behavioral treatment for severe feeding problems: A cost‐effective alternative to tube feeding? Journal of Developmental and Physical Disabilities, 19(3), 227–235. 10.1007/s10882-007-9051-y

[jcv212160-bib-0103] World Health Organization . (2019). International statistical classification of diseases and related health problems (11th ed.). https://icd.who.int/ 10.2471/BLT.25.294650 PMC13531092 42683092

[jcv212160-bib-0104] Yelencich, E. , Truong, E. , Widaman, A. M. , Pignotti, G. , Yang, L. , Jeon, Y. , Weber, A. T. , Shah, R. , Smith, J. , Sauk, J. S. , & Limketkai, B. N. (2022). Avoidant restrictive food intake disorder prevalent among patients with inflammatory bowel disease. Clinical Gastroenterology and Hepatology, 20(6), 1282–1289.e1281. 10.1016/j.cgh.2021.08.009 34389486

[jcv212160-bib-0105] Yule, S. , Wanik, J. , Holm, E. M. , Bruder, M. B. , Shanley, E. , Sherman, C. Q. , Fitterman, M. , Lerner, J. , Marcello, M. , Parenchuck, N. , Roman‐White, C. , & Ziff, M. (2021). Nutritional deficiency disease secondary to ARFID symptoms associated with autism and the broad autism phenotype: A qualitative systematic review of case reports and case series. Journal of the Academy of Nutrition and Dietetics, 121(3), 467–492. 10.1016/j.jand.2020.10.017 33221247

[jcv212160-bib-0106] Zickgraf, H. F. , & Ellis, J. M. (2018). Initial validation of the nine item avoidant/restrictive food intake disorder screen (NIAS): A measure of three restrictive eating patterns. Appetite, 123, 32–42. 10.1016/j.appet.2017.11.111 29208483

[jcv212160-bib-0107] Zickgraf, H. F. , Lane‐Loney, S. , Essayli, J. H. , & Ornstein, R. M. (2019). Further support for diagnostically meaningful ARFID symptom presentations in an adolescent medicine partial hospitalization program. International Journal of Eating Disorders, 52(4), 402–409. 10.1002/eat.23016 30632634 PMC7057554

[jcv212160-bib-0108] Zickgraf, H. F. , Murray, H. B. , Kratz, H. E. , & Franklin, M. E. (2019). Characteristics of outpatients diagnosed with the selective/neophobic presentation of avoidant/restrictive food intake disorder. International Journal of Eating Disorders, 52(4), 367–437. 10.1002/eat.23013 30636013 PMC8056744

[jcv212160-bib-0109] Zimmerman, J. , & Fisher, M. (2017). Avoidant/restrictive food intake disorder (ARFID). Current Problems in Pediatric and Adolescent Health Care, 47(4), 95–103. 10.1016/j.cppeds.2017.02.005 28532967

